# Controlled Release of Mesenchymal Stem Cell-Conditioned Media from a Microsphere/Gel-Based Drug Delivery System for Wound Healing of Tympanic Membrane Perforations

**DOI:** 10.1155/2023/6039254

**Published:** 2023-12-31

**Authors:** Liza A. Bruk, Xin Fan, Jayde L. Resnick, Morgan V. DiLeo

**Affiliations:** ^1^Department of Bioengineering, University of Pittsburgh, 3501 Fifth Ave, Pittsburgh, PA 15213, USA; ^2^Department of Ophthalmology, University of Pittsburgh, 3501 Fifth Ave, Pittsburgh, PA 15213, USA; ^3^Department of Clinical and Translational Science, University of Pittsburgh, 3600 Forbes Ave, Pittsburgh, PA 15213, USA; ^4^Department of Chemical Engineering, University of Pittsburgh, 3501 Fifth Ave, Pittsburgh, PA 15213, USA

## Abstract

Chronic tympanic membrane (TM) perforation increases patient susceptibility to infection, hearing loss, and other side effects. Current clinical treatment, surgical grafting, can result in detrimental side effects including nerve damage, dizziness, or hearing loss. Therefore, it is essential to develop novel therapeutic procedures that can induce or accelerate healing in minimally or noninvasive approaches. Cell-free therapies have safety advantages over stem cells and are logistically favorable for clinical use. The regenerative potential by mesenchymal stem cell-conditioned media (CM) has been promising. In this study, poly(lactic-co-glycolic acid) (PLGA) microspheres with CM encapsulated have been developed as a cell-free alternative regenerative treatment for TM perforation. The results suggest that the PLGA microspheres were capable of encapsulating and releasing CM for up to 21 days. The *in vitro* scratch wound proliferation assays showed increased wound healing ability of CM-loaded microspheres. *In vivo* guinea pig models treated with CM drops and CM-loaded microspheres using a thermoresponsive gel carrier demonstrated potential for wound healing in TM perforation. These studies provide a basis for further examination of the delivery of stem cell CM and investigation of time-dependent wound healing, long-term ototoxicity, and hearing restoration.

## 1. Introduction

Perforation of the tympanic membrane (TM) can result from various causes, including infections, trauma, and iatrogenic injury. Most perforations could heal spontaneously; however, some could become chronic. Chronic TM perforations persist for more than three months and require surgical treatment, such as myringoplasty or tympanoplasty, utilizing a graft or synthetic material to control infection and enhance hearing [1, 2]. However, these procedures are expensive, time consuming, and carry the risk of nerve damage, hearing loss, tinnitus, or dizziness [3, 4]. Studies indicated that more than 88% of the subjects with chronic perforations were opposed to the idea of surgical grafting [5]. Consequently, the development of innovative, minimally or noninvasive treatment options that can induce or accelerate healing would significantly enhance outcomes for individuals with TM perforations.

Tissue engineering holds promise for repairing TM perforation using regenerative medicine techniques that employ biomaterials, growth factors, or stem cells [6]. Natural and synthetic biomaterials, such as hyaluronic acid, collagen, cellulose, and polylactic acid, have been extensively investigated for treating TM perforations [7–10]. The most commonly used growth factors for TM regeneration are the epidermal growth factor (EGF) and the basic fibroblast growth factor (bFGF) [11]. The use of bFGF has been reported to modulate the rate of healing for small perforations (≤25% of the total TM area) in clinical studies [12]. Stem cells have also been used for TM repair through the topical application or combined with biomaterial scaffolds. Stem cells improve tissue regeneration by releasing trophic factors, cytokines, matrix proteins, or differentiating into local tissue [13]. However, due to the in-air suspension of the TM tissue, stem cell application or scaffold materials are prone to evaporative drying, which may not sustain cell viability long enough for healing to occur [14]. Cell therapy presents additional logistical and regulatory hurdles including scale-up and distribution [15].

Stem cell conditioned media (CM), also known as secretome, has emerged as a promising cell-free alternative for regenerative medicine. CM includes molecules and biological factors secreted by cells into the extracellular space, such as soluble factors (growth factors, cytokines, chemokines, and enzymes) and extracellular vesicles that transport lipids, proteins, and RNAs [16]. Cell-free CM-based therapies offer biological benefits and reduce safety concerns of stem cells while providing logistical advantages for clinical use, including scalability, availability, and longer shelf-life [17]. Several groups have demonstrated that the CM from bone marrow mesenchymal stem cell (MSC) cultures share similar properties with MSCs themselves *in vivo*, suggesting that the paracrine/soluble factors released by MSCs play a significant regenerative role [18, 19]. To enhance therapeutic efficiency, delivery systems and strategies have been developed to provide sustained and controllable delivery of stem cell CM [20]. However, to the best of our knowledge, no sustained stem cell CM delivery system has been studied for TM perforation.

This study aimed to evaluate the feasibility of a poly(lactic-co-glycolic acid) (PLGA) microsphere (MS)-based drug delivery system for CM. Our group has previously developed similar controlled release systems for the treatment of various ocular and otic conditions [21–24]. In this study, we propose the use of stem cell CM-loaded MS with a topical thermoresponsive gel depot to promote wound healing. We hypothesize that the encapsulation and controlled release of CM from PLGA MS will safely promote wound healing for chronic TM perforation, providing a similar effect as stem cell therapies that may require complicated scaffold matrix systems.

## 2. Materials and Methods

### 2.1. Mesenchymal Stem Cell-Conditioned Media Preparation

Adipose derived mesenchymal stem cells (MSCs) (RoosterBio Inc.) were cultured in T175 flasks in proprietary RoosterNourish MSC media (RoosterBio). Source donors were adults under 45 years to avoid potential deficits in MSC from more elderly patients [25–27]. When flasks reached ∼70% confluence, 20 mL of fresh media was added and cells were allowed to culture for 48 hours. The media was removed and centrifuged at 1200 g for 5 minutes to remove any cell debris, flash frozen, and stored at −20°C. The remaining cells were subcultured, and the process was repeated until passage 3. CM was then filtered in 10 kDa filter tubes (Amicon Ultra, MilliporeSigma, Burlingame, MA), frozen, lyophilized, and reconstituted in 0.45 mL deionized (DI) water. The concentrations of total protein and the fibroblast growth factor (FGF-2) in CM were quantified using MicroBCA Protein Assay and Invitrogen Human FGF ELISA kits (Thermo Fisher Scientific, Waltham, MA), respectively.

### 2.2. Microsphere Fabrication and Characterization

CM-loaded PLGA microspheres (CM-MSs) were prepared using a water-in-oil-in-water double emulsion procedure adapted from our previous work [21, 22, 28]. In brief, 200 mg PLGA (MW: 38–54 kDa; viscosity: 0.56–0.6 dL/g, MilliporeSigma, Burlingame, MA) were dissolved in 4 mL dichloromethane to which 200 *µ*L of reconstituted CM was added. The dissolved drug and polymer mixture were then sonicated for 10 seconds at 30% amplitude (EpiShear Probe Sonicator, Active Motif, Carlsbad, CA), followed by homogenization in 60 mL of 2% poly (vinyl alcohol) (PVA) (Polysciences, Warrington, PA) for 1 minute at 7000 rpm (Silverson L5M-A, East Longmeadow, MA). The resulting liquid-phase emulsion was added to 80 mL of 1% PVA and stirred at 600 rpm for 3 hours, resulting in the precipitation of solid CM-MS. CM-MSs were then washed 4 times by centrifugation, resuspended in DI water, flash frozen in liquid nitrogen, and lyophilized for 48–72 hours (Benchtop Pro, SP Scientific, Warminster, PA). Blank MSs were fabricated by substituting DI water for reconstituted CM. Scanning electron microscopy (SEM) was used to examine shape and morphology of CM-MS (JEOL JSM 6335F, Peabody, MA).

### 2.3. *In Vitro* Release Study

*In vitro* drug release kinetics were determined using 10 mg of MS suspended in 0.5 mL phosphate buffered saline (PBS) and continuously rotated at 37°C. The supernatant was removed via centrifugation every 24 hours and replaced with 0.5 mL fresh PBS. Total protein concentration in the supernatant was quantified by the MicroBCA Protein Assay kit (Thermo Fisher Scientific, Rockford, IL). UV/Vis absorbance was measured at 562 nm (SoftMax Pro 5, Molecular Devices, Sunnyvale, CA) with background signal from blank MS subtracted from each measurement and regressed against the standard curve. FGF-2 concentration was quantified via the Human FGF ELISA kit (Invitrogen, Carlsbad, CA).

### 2.4. Scratch Wound Proliferation Assay

The scratch wound assay method was adapted from Walter et al. [29]. Human primary epidermal keratinocytes were incubated in the dermal cell basal medium using the Keratinocyte Growth Kit at 37°C with 5% CO_2_ until flasks reach 70–80% confluency and then transferred to 24-well plate at a density of ∼65,000 cells/well. Cells were incubated for 24–48 h in 1 mL basal medium until a confluent monolayer was achieved. Vertical scratch was accomplished using a 1 mL pipette tip and wells were rinsed 1-2 times with the medium to remove any dislodged cells. Scratch wounds were then immediately treated, in quadruplicate, with (a) 1 mL basal medium, (b) 1 mL MSC CM, (c) blank MS releasates, and (d) CM-MS releasates. MSC CM was processed via filtration for 20 mins at 4000 rpm in 3 kDa filter tubes (Amicon Ultra, MilliporeSigma, Burlingame, MA) and diluted with DI water to approximate release from CM-MS. To prepare MS releasates, 30 mg MS were suspended in 1 mL basal medium and rotated at 37°C for 24 h prior to centrifugation and filtration at 8200 rpm for 30 mins in 10 kDa ultra centrifugal filters (Amicon, MilliporeSigma, Cork, Ireland), and the filtrated release medium was used as the MS releasates. Images were collected at time points of 0, 6, 12, 24, and 48 h after treatment using an inverted microscope at 10x magnification (Leica Microsystems DMi1, Buffalo Grove, IL) and digital camera (Canon Powershot G7X Mark II, Tokyo, Japan). Images were analyzed using ImageJ software using the MRI wound healing tool to determine the scratch wound area at each time point, with data presented as wound healing percentage compared to *t* = 0 h scratch wound area.

### 2.5. *In Vivo* Chronic Tympanic Membrane Perforation and Treatment

**Figure 1:**
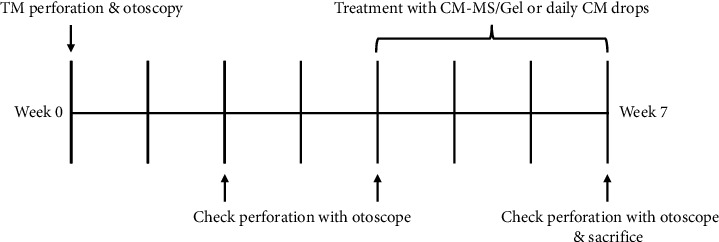
Timeline of *in vivo* TM perforation studies in the guinea pig model. CM, conditioned media; CM-MSs, CM-loaded PLGA microspheres.

The animal protocol for this study was reviewed and accepted by the University of Pittsburgh Institutional Animal Care and Use Committee. Eighteen male Dunkin Hartley guinea pigs (8 weeks, ∼500 g) were purchased from Charles River. All experiments were performed under 1-2% isoflurane anesthesia.  Figure 1 shows the timeline of imaging and treatment for this study. Perforation was achieved under anesthesia using an 18 G needle. TMs were imaged using video otoendoscopy (Karl Storz vetcam XL with xenon nova 175 light source, Edgewater, MD) before and after perforation. Mitomycin C (Alfa Aesar, Thermo Fisher Scientific, Ward Hill, MA) was applied at a concentration of 0.5 mg/mL via gelfoam sponge for 5 mins after perforation. Perforation was followed by twice-daily treatment with 1% hydrocortisone (Zymox, PKB Animal Health, Westmont, IL) and 0.2% ciprofloxacin (Sigma-Aldrich, St. Louis, MO) for 7 days and subsequently 3 undisturbed weeks allowing perforation to develop as confirmed by otoendoscopy. For each otoscopic analysis, any ear wax or dried blood in the ear canal were cleared using a cotton-tipped applicator and/or lavage with sterile water as needed. Four weeks after perforation, animals were randomized into one of the three treatment groups, with *n* = 6 animals per group as follows: no treatment, treatment with daily MSC CM topical drops, and treatment with CM-MS/gel. MSC CM was processed via filtration for 20 mins at 4000 rpm in 3 kDa filter tubes (Amicon Ultra, MilliporeSigma, Burlingame, MA) and resulting filtrate diluted with DI water to approximate release from CM-MS. Approximately 100 *µ*L of these drops were applied at the same time every day for 21 days. For the CM-MS/Gel treatment group, 200 mg CM-MS were mixed with 2 mL gel and each animal received 100 *µ*L of the mixture administered via 1 mL syringe. The reverse thermoresponsive gel used has been previously fabricated and extensively characterized [21, 22]. In brief, the gel was prepared via free radical polymerization of N-isopropylacrylamide (NIPAAm) (Fisher Scientific, Waltham, MA) and poly(ethylene glycol) (MW ∼200 kDa) in the presence of ammonium persulfate and tetramethylethylenediamine. After 24 h refrigeration, gel precursor was washed five times in DI water at ∼40°C and stored at 4°C until use.

### 2.6. Histopathology

After 3 weeks of treatment, animals were euthanized via intracardiac sodium pentobarbital under isoflurane anesthesia. TMs were excised and fixed in 10% formalin for 24 hours. TMs were then dehydrated overnight in 70% ethanol, embedded in paraffin, sectioned in 5 *µ*m thick sections, and stained with hematoxylin and eosin (H & E). Stained sections were imaged and evaluated using light microscopy (Leica Microsystems DM2500) and digital microscope camera (Leica Microsystems DFC295) by a blinded technician.

### 2.7. Statistical Methods

Data are represented as the average ± standard deviation for *n* = 3 samples for *in vitro* release assays. Wound healing percentages in scratch assay studies were analyzed using one-way analysis of variance (ANOVA) followed by Dunnett's multiple comparisons test (GraphPad Prism 10; GraphPad Software, San Diego, CA) comparing scratch wounds treated with MSC CM, blank MS releasates, and CM-MS releasates to basal medium at each time point separately. Significance was established when the *p* value was less than 0.05.

## 3. Results

### 3.1. *In Vitro* Release Study

**Figure 2:**
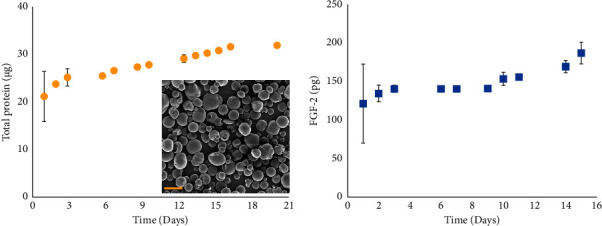
*In vitro* conditioned media release from CM-MS. (a) Cumulative total protein release from 10 mg CM-MS. Inset: scanning electron microscopy images of CM-MS (scale bar = 10 *µ*m). (b) Cumulative FGF-2 release from 10 mg CM-MS.

The spherical morphology of the CM-MS was confirmed by scanning electron microscopy (Figure 2(a)). The release of conditioned media from the microspheres demonstrated a burst release pattern followed by approximately linear release. Cumulative release of total protein (Figure 2(a)) showed that 21 *µ*g of total protein was released on day 1, followed by an additional 10 *µ*g released over the next 20 days. A similar release pattern was observed for FGF-2 (Figure 2(b)), with 121 pg of FGF-2 released on day 1 and an additional 75 pg released over the next 14 days.

### 3.2. Scratch Wound Assays

**Figure 3:**
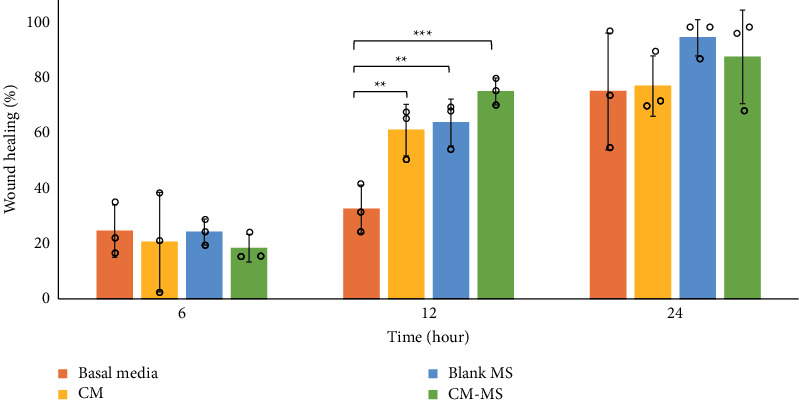
Scratch wound healing over time. Wound healing percentages at *t* = 6, 12, and 24 h after scratch, determined by comparing scratch wound area at each time point to *t* = 0 h using ImageJ software. Error bars represent the mean ± standard deviation for *n* = 3 samples for all groups. Significance (^*∗∗*^*p* < 0.01 and ^*∗∗∗*^*p* < 0.001) was determined by one-way ANOVA followed by Dunnett's multiple comparisons test. CM, conditioned media; CM-MSs, CM-loaded PLGA microspheres.

**Figure 4:**
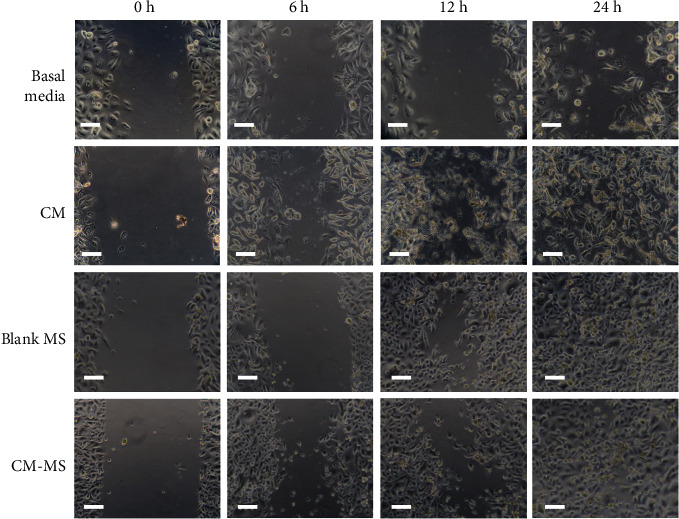
Scratch wound assay images. Representative digital images of scratch wounds in human primary dermal keratinocytes cultured in 24-well plates at time 0, 6, 12, and 24 h. Scratch wounds were incubated in dermal basal media, CM, releasates from blank MS, and releasates from CM-MS. Images were analyzed for scratch wound area using ImageJ. Scale bar = 100 *µ*m. CM, conditioned media; CM-MSs, CM-loaded PLGA microspheres.

The wound healing percentages were determined by measuring the scratch wound area at each time point relative to the baseline at *t* = 0 h (Figure 3). At *t* = 6 h, no significant differences were observed among the groups, with all average wound healing percentages below 25%. At *t* = 12 h, a significant increase in wound healing (*p* < 0.05) was observed for the conditioned media, CM-MS, and blank MS treatment groups compared to basal media. The CM-MS group exhibited the highest wound healing percentage (76%). At *t* = 24 h, no significant differences were observed among the groups, with all average wound healing percentages greater than 70%. The CM-MS treatment resulted in 89% wound healing. By *t* = 48 h, all scratch wounds were completely healed.  Figure 4 shows the representative images of scratch wound healing per group over time.

### 3.3. *In Vivo* Chronic TM Perforation and Treatment

**Figure 5:**
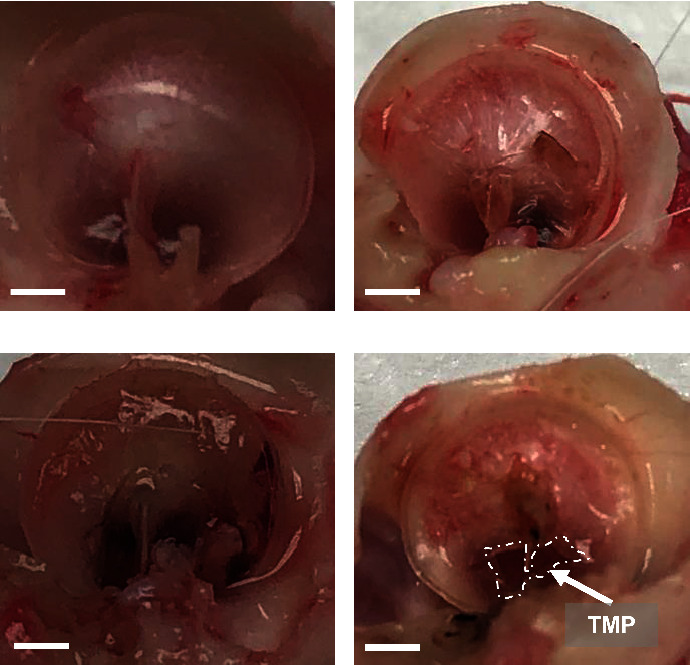
Representative images of TM postsacrifice. (a) Pristine TM from contralateral ear with no manipulations or treatment; (b) healed perforation and normal physiology characterized by sagging TM and presence of dried blood; (c) healed perforation but abnormal physiology; (d) residual perforation. The white arrow pinpoints the location of the perforation. TMP, tympanic membrane perforation. Scale bar = 60 mm.

**Figure 6:**
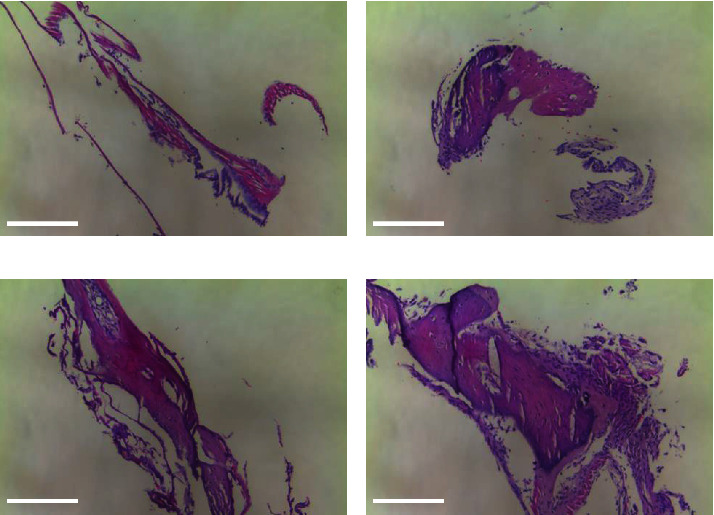
Representative images of H & E-stained guinea pig TMs. Images represent (a) pristine TM; (b) healed perforation and normal physiology; (c) healed perforation but abnormal physiology; (d) residual perforation. Scale bars = 200 nm.

After sacrifice, both the left and right TMs were removed from each animal, visually inspected, and photographed to determine the presence of perforation or any abnormal physiology. As expected, all left ears, which had no manipulations or treatment, were pristine (Figure 5(a)). The right ears were classified into three categories as follows: (1) healed perforation and normal physiology (Figure 5(b)); (2) healed perforation but abnormal physiology (Figure 5(c)); and (3) residual perforation (Figure 5(d)). While the tissue samples for pristine TM (Figure 6(a)) and TM with healed perforation and normal physiology (Figure 6(b)) got folded during dissection and staining, H & E staining indicates normal physiology and lack of edema or immune response, comparable to healthy guinea pig TMs. However, TM with healed perforation but abnormal physiology (Figure 6(c)) exhibited increased edema. TM with residual perforation showed increased edema and enlargement compared to other samples, indicating damage and possible infection due to perforation (Figure 6(d)).

**Figure 7:**
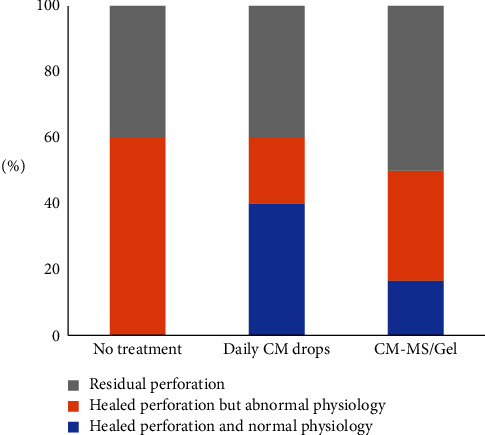
Observation of TM perforation in guinea pigs after 21 days of different treatments. CM, conditioned media; CM-MSs, CM-loaded PLGA microspheres.

For observation of TM perforation in guinea pigs after 21 days in different treatments, Figure 7 represents the ratios of different statues of TM perforations by different treatment groups. In the no-treatment group, 2 of 5 ears had residual perforation (40%), with the remaining 3 having healed perforation but abnormal physiology (60%), including sagging rather than tight TM and the presence of blood or other fluids. None of the ears showed a healed perforation or normal physiology. In the group treated with daily CM drops, 2 of 5 ears also had residual perforation (40%); however, 2 had healed perforation and normal physiology (40%) and 1 had healed perforation but abnormal physiology (20%), suggesting increased wound healing due to the application of conditioned media. In both groups, *n* = 5 animals were analyzed, as one animal from each group was removed from the study. One animal was removed from the no-treatment group due to an adverse reaction to anesthesia, and one was removed from the CM drops group due to an infection unrelated to treatment.

**Figure 8:**
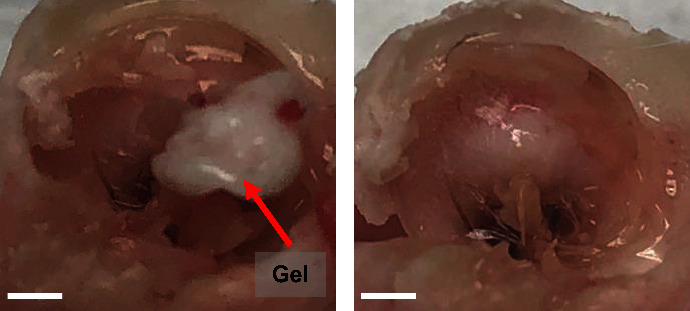
CM-MS/gel after sacrifice and dissection: (a) on reverse side of TM inside bulla and (b) same TM but with gel removed showing lack of TM perforation but abnormal physiology. Scale bar = 60 mm.

In the group treated with CM-MS/Gel, gel was found inside the bulla (Figure 8(a)), indicating either that it was placed through the TM perforation during instillation or sunk through the perforation into the bulla during treatment. When the gel was placed on the reverse side of the TM, healed TM was visualized after the removal of the gel (Figure 8(b)). The presence of gel physically blocking the TM perforation from healing may have had an adverse effect on wound healing, with 3 of the 6 animals having residual perforation (50%) and abnormal physiology including gel protruding through the TM perforation and fluid and cerumen buildup. However, 1 animal had healed perforation with normal physiology (20%) and 2 animals had healed perforation but with some abnormal physiology (40%) including fluid and cerumen buildup. All 6 animals in this group were analyzed.

## 4. Discussion

The surgical treatment of chronic TM perforations can take up to three months to achieve complete healing, during which patients may experience infection, tinnitus, vertigo, or loss of hearing, leading to a temporary or permanent negative impact on their quality of life [4]. To address these limitations, our group developed a PLGA microsphere-based controlled delivery system to encapsulate and deliver MSC CM for tympanic membrane perforations.

The *in vitro* release results demonstrated that CM-MSs were effective in controlling the release of total protein over a period of 21 days. CM-MS released total protein in a controlled manner, with a 66% burst release on day 1 followed by a 20-day sustained release. This study demonstrates the potential of PLGA MS as a sustained drug delivery platform for CM. Encapsulation and release of FGF-2 were of particular interest for wound healing of tympanic membrane perforations. CM-MSs were able to reach a cumulative release of 206.5 ± 14.3 pg of FGF-2 released from 10 mg of CM-MS (Figure 2(b)). Previous studies have demonstrated a range of FGF-2 concentration to be effective for cell proliferation purposes, including an *in vitro* study suggesting that 14 pg/mm^2^ in 3D printed fibrin substrates increased cell density on the substrates and lengthened time of cell survival [30]. Another study found that 1 *μ*g of bFGF encapsulated in microspheres and embedded in collagen scaffolds improved cell seeding of intestinal smooth muscle cells [31]. For TM perforation healing in particular, studies have shown that daily doses of approximately 80 ng bFGF improved wound healing time and decreased otorrhea [32, 33]. These cited values are mostly greater than the release seen in our MS formulation. However, further investigation is needed into vesicle shedding on each day of MS release and amount of FGF bound in and released from these vesicles.

Scratch wound proliferation assays further suggested the safety of CM and CM-MS. No detrimental effect was observed due to treatment with CM, blank MS, and CM-MS. Significantly increased proliferation at *t* = 12 h due to culturing scratch wound in mesenchymal stem cell CM compared to basal media confirmed previous results reported by Walter et al. [29].

For observation of TM perforation in guinea pigs, the outer diameter of an 18 G needle is 1.27 mm, which results in a surface area of 1.27 mm^2^ based on the standard surface area equation for a circle, *A*=*πr*^2^. The guinea pig TM diameter of approximately 2.5 mm yields surface area of 4.91 mm^2^; therefore, an 18 G needle should result in perforation of approximately 25% of the TM surface area. The group that received no treatment resulted in 40% persistent TM perforation, which is consistent with previously cited statistics of 6–46% of TM perforations being nonhealing without treatment [33, 34]. The remaining 60% displayed abnormal physiology including buildup of fluid, dried blood, and cerumen, indicating incomplete healing despite the lack of residual TM perforation. Although the group treated with daily CM drops also resulted in 40% nonhealing, only 20% had abnormal physiology and the remaining 40% had no residual TM perforation and normal physiology. These results suggest a positive effect on wound healing due to the application of conditioned media drops, which is expected due to previous studies on the growth factor and stem cell treatment exhibiting positive effects on wound healing [33, 35–39]. All CM-MS/gel systems migrated through the TM perforation into the bulla either during placement or treatment. In several cases, the CM-MS/gel system protruded through the TM perforation and physically prevented wound healing. This may also have contributed to the increased fluid and cerumen buildup, as abnormal physiology was observed and 33.3% of the ears showed no residual perforation but persistent abnormalities. However, despite gel/MS migration into the bullae, 16.7% of the ears had complete wound healing.

There are a few limitations to the guinea pig TM perforation model described herein that can be addressed in future studies. Perforation was achieved using an 18 G needle in this study, which occasionally resulted in injury to the external ear canal, causing blood and subsequently dried blood to confound imaging. Furthermore, this technique may have caused a tear rather than a distinct perforation of the TM and results in difference sizes of the perforations. To ensure precise control of the perforation, a myringotomy needle attachment can be used in conjunction with the otoscope. Irrigation and suction attachments can be used to better clear ear canal of any ear wax, blood, or moisture that can confound visualization and imaging. Myringotomy can also be performed repeatedly over several weeks to ensure that perforation becomes chronic [40]. In conjunction with otoendoscopic imaging for qualitative assessment of TM wound healing, auditory brainstem responses can be used as described previously to confirm perforation due to conductive hearing loss because of nonintact TM and confirm adequate wound healing determined by a renewed capacity to hear using injured ear [41–43]. To determine the positive effect more accurately on wound healing due to topical CM drops and CM-MS/gel treatment, auditory brainstem responses can be used longitudinally in addition to imaging to track renewed hearing sensitivity after perforation and during treatment, as well as at a time point of some weeks or months after treatment to determine long-term effects of treatment.

## 5. Conclusion

This study suggests that the PLGA MS system is capable of encapsulating and subsequently releasing MSC CM for up to 21 days. Further investigation is necessary to determine daily vesicle shedding from CM-MS and total protein and FGF-2 content in these vesicles. *In vitro* scratch wound proliferation assays suggest that these materials can be safe for *in vivo* and clinical use and indicate wound healing ability. Although there were limitations in the TM perforation animal model, there is evidence to suggest that CM has a positive impact on wound healing and CM-MS/gel placement, with some improvement and refinement of placement and removal techniques, may also have a positive effect on wound healing. Future studies are warranted to investigate long-term ototoxicity and time-dependent effects on hearing sensitivity using auditory brainstem response testing.

## Data Availability

The data used to support the findings of this study are available from the corresponding author upon reasonable request.
